# Autophagy attenuates endothelial-to-mesenchymal transition by promoting Snail degradation in human cardiac microvascular endothelial cells

**DOI:** 10.1042/BSR20171049

**Published:** 2017-09-07

**Authors:** Jin Zou, Yanhua Liu, Bingong Li, Zeqi Zheng, Xuan Ke, Yanqin Hao, Xuelian Li, Xingxing Li, Fuyou Liu, Zhiyong Zhang

**Affiliations:** 1 Department of Cardiology, First Affiliated Hospital, Nanchang University, Nanchang 330006, Jiangxi Province, China; 2 Department of Cardiology, Loudi Central Hospital, Loudi 417000, Hunan Province, China

**Keywords:** autophagy, hypoxia, ndothelial-to-mensenchymal transition, Snail, TGFβ2

## Abstract

Endothelial-to-mesenchymal transition (EndMT) mainly exists in cardiovascular development and disease progression, and is well known to contribute to cardiac fibrosis. Recent studies indicated that autophagy also participates in the regulation of cardiac fibrosis. However, the precise role of autophagy in cardiac fibrosis and the underlying molecular mechanism remain unclear. The present study aimed to explore the role of autophagy in EndMT, reveal the underlying molecular mechanism, and seek new therapy for cardiac fibrosis. In the present study, we found that EndMT and autophagy were induced simultaneously by hypoxia in human cardiac microvascular endothelial cells (HCMECs). Rapamycin, an autophagy enhancer, attenuated EndMT with promoting angiogenesis, while 3-methyladenine (3-MA) and chloroquine (CQ), agents that inhibit autophagy, accelerated the progression accompanied by the decrease in counts of tube formation under hypoxia conditions. Interestingly, intervening autophagy by rapamycin, 3-MA, or CQ did not affect hypoxia-induced autocrine TGFβ signaling, but changed the expression of Snail protein without alterations in the expression of *Snail* mRNA. Furthermore, the colocalization of LC3 and Snail indicated that autophagy might mediate Snail degradation under hypoxia conditions in HCMECs. Interaction of p62, the substrate of autophagy, with Snail by co-immunoprecipitation especially in hypoxia-incubated cells confirmed the hypothesis. In conclusion, autophagy serves as a cytoprotective mechanism against EndMT to promote angiogenesis by degrading Snail under hypoxia conditions, suggesting that autophagy targetted therapeutic strategies may be applicable for cardiac fibrosis by EndMT.

## Introduction

Cardiac fibrosis is the primary pathologic hallmark of ventricular remodeling, which leads to heart failure, arrhythmias, dilated cardiomyopathy, and sudden death [1,2]. Thus, it is of clinical significance to prevent and reverse myocardial fibrosis. The primary pathological process of cardiac fibrosis is the proliferation and activation of myocardial fibroblasts, which contribute to the excessive accumulation of extracellular matrix (ECM) [3]. Excessive proliferation of myocardial fibroblasts and production of ECM decrease the elasticity of ventricular wall while increasing the stiffness and reducing ventricular wall motion [4]. Recent studies have suggested that myocardial fibroblasts originate from resident fibroblasts, bone marrow derived fibroblasts, epithelial cells by undergoing epithelial-to-mesenchymal transition (EMT), and endothelial cells (ECs) by undergoing endothelial-to-mesenchymal transition (EndMT) [5,6]. The process of EndMT, similar to that of EMT, is characterized by the loss of EC makers such as E-cadherin and PECAM-1/CD31, and the acquiring of mesenchymal phenotypes such as α-smooth muscle actin (α-SMA), fibroblast protein-1 (FSP1), and type 1 collagen (COL1) [7]. EndMT has been reported in a variety of physiological and pathological phenomena. For example, during heart embryonic development, ECs from endocardium can give rise to the valves and septa of the heart via EndMT [8,9]. Under the pathological condition, EndMT contributes to many tissue fibrosis diseases such as pulmonary arterial hypertension (PAH) [10], systemic sclerosis (SSc) [11], and unilateral ureteral obstruction (UUO) [12]. In particular, EndMT is involved in the development of cardiac fibrosis diseases, such as acute myocardial infarction (AMI) [13], heart failure [14], and hypertension [15]. It has been shown that the elevation of exogenous and endogenous TGFβ is a potent inducer of EndMT [16], but accumulating evidence have also suggested that hypoxia [17], inflammation [18], and high glucose [19] also induce EndMT. The precise molecular mechanism underlying EndMT is yet to be fully elucidated.

Autophagy is a conserved process in which eukaryotic cells deliver the damaged, degenerative, and aged proteins and organelles into lysosome for digestion and degradation to maintain eukaryotic self-stability [20]. Under the physiological condition, autophagy degrades cellular constituents to provide nutrition and materials for recycling. Under the pathological conditions (including starvation, hypoxia, inflammation, and oxidative stress), autophagy acts as an adaptive response to external stress to maintain the overall balance. Autophagy plays an important role in the pathophysiology of many diseases such as cancer, neurodegenerative diseases, ageing, and cardiovascular diseases [21,22]. It has been reported that autophagy modulates the progression of cardiac fibrosis [23,24]. Meanwhile, the role of EndMT in promoting cardiac fibrosis has been demonstrated. Several studies have indicated that autophagy also has an important effect on the process of EMT [25,26], but its role in EndMT has rarely been reported. Therefore, we hypothesized that intervening autophagy may affect the occurrence of EndMT and even cardiac fibrosis.

Our recent study has reported that hypoxia induces EndMT in human cardiac microvascular ECs (HCMECs), during which TGF-β signaling pathway is activated [27]. In the present study, we confirmed the findings and demonstrated that autophagy exerts cytoprotection against EndMT in HCMECs to promote tube formation under hypoxia by mediating Snail degradation. This may provide an updated understanding of underlying molecular mechanisms of EndMT.

## Materials and methods

### Cell culture and treatment

HCMECs were purchased from Sciencell Research Laboratories and maintained in EC medium (Sciencell, California, U.S.A.) as described recently [27]. A density of 10000 cells/cm^2^ of passages 2–6 were seeded in a six-well plate. Cells were divided into five groups including – (i) control group: HCMECs were cultured under normoxia conditions; (ii) hypoxia group: HCMECs were cultured under hypoxia conditions (1% O_2_) for 3 days; (iii) hypoxia + rapamycin group: HCMECs were pretreated with rapamycin (100 nM, Sigma–Aldrich, St. Louis, MO, U.S.A.) for 2 h and cultured under hypoxia conditions (1% O_2_) for 3 days; (iv) hypoxia + 3-methyladenine (3-MA) group: HCMECs were pretreated with 3-MA (5 mM, Sigma–Aldrich, St. Louis, MO, U.S.A.) for 2 h and cultured under hypoxia conditions (1% O_2_) for 3 days; and (v) hypoxia + chloroquine (CQ) group: HCMECs were pretreated with CQ (20 μM, Sigma–Aldrich, St. Louis, MO, U.S.A.) for 2 h and cultured under hypoxia conditions (1% O_2_) for 3 days.

### Western blot

Cells were homogenized in RIPA lysis buffer (Solarbio, Beijing, China) containing protease inhibitor cocktail (Roche, Mannheim, Germany) on ice. After the mixture was centrifuged at 12000***g*** for 10 min at 4°C, the supernatant was collected. The protein concentration was measured using BCA Protein Assay Kit (Beyotime, Beijing, China) according to the manufacturer’s protocol. Proteins were separated by SDS/PAGE and then transferred on to PVDF or NC membranes (Solarbio, Beijing, China). The membranes were blocked in TBST (TBS with 0.1% Tween 20, pH: 7.2) containing 5% non-fat milk (Solarbio, Beijing, China) for 1 h, then incubated with primary antibodies (Table 2), followed by HRP–conjugated secondary antibodies (Table 3) at room temperature. Proteins were detected using the chemiluminescent kit (TransGen Biotech, Beijing, China).

### qRT-PCR

Total RNAs were extracted from ECs using TRIzol reagent (Life Technologies, New York, U.S.A.) according to the manufacturer’s protocol. One microgram of RNA was reverse transcribed into cDNA using cDNA Synthesis Kit (Promega, Beijing, China) using the ThermoScript Reverse Transcription System (Thermo Fisher Scientific) for RT-PCR reaction. β-actin was used as the endogenous control and the primer sequences (Generay, Shanghai, China) are listed in Table 1.RT-PCR was performed on ABI 7500 Real-Time PCR System (Applied Biosystems, Carlsbad, U.S.A.) using GoScript™ SYBR-Green PCR Kit (Promega, Beijing, China) according to the manufacturer’s instructions. Relative expression of mRNAs of interest was analyzed using the 2^−ΔΔt^ method as recently described [28].

**Table 1:** RT-PCR primer sequences

Gene	Forward (5′ to 3′)	Reverse (5′ to 3′)
*CD31*	CCCAGCCCAGGATTTCTTAT	ACCGCAGGATCATTTGAGTT
*E-cadherin*	CCAATCTGACCTGAAAAAGC	CCACCGTTTTCCGTGTAATA
*α-SMA*	AAGCACAGAGAGCAAAAGAGGAAT	ATGTCGTCCCAGTTGGTGAT
*FSP1*	AACTAAAGGAGCTGCTGACCC	TGTTGCTGTCCAAGTTGCTC
*Snail*	AATCGGAAGCCTAACTACAGCGAG	CCTTGGCCTCAGAGAGCTGG
*β-actin*	GCCATGTACGTAGCCATCCA	GAACCGCTCATTGCCGATAG
*TGFβ2*	TACGCCAAGGAGGTTTACAAA	TGAAGTAGGGTCTGTAGAAAGTG
*Smad2*	GGAGCAGAATACCGAAGGCA	CTTGAGCAACGCACTGAAGG
*Smad3*	ATTCCAGAAACGCCACCTCC	GCTATTGAACACCAAAATGCAGG

**Table 2:** Primary antibodies

Primary antibody	Company	Dilute solution	Dilution rate
Mouse anti-CD31	Cell Signaling	2% non-fat milk	1:1000
Rabbit anti-E-cadherin	Cell Signaling	2% non-fat milk	1:1000
Rabbit anti-α-SMA	Abcam	2% non-fat milk	1:5000
Rabbit anti-FSP1	Abcam	2% non-fat milk	1:2000
Rabbit anti-Snail	Abcam	2% non-fat milk	1:2000
Mouse anti-β-actin	Zhongshan Golden Bridge Biotechnology	2% non-fat milk	1:10000
Rabbit anti-LC3	Cell Signaling	2% non-fat milk	1:1000
Rabbit anti-beclin1	Cell Signaling	2% non-fat milk	1:1000
Rabbit anti-p62	Cell Signaling	2% non-fat milk	1:1000
Rabbit anti-TGFβ2	Abcam	2% non-fat milk	1:1000
Rabbit anti-Smad2	Bioss	2% non-fat milk	1:500
Rabbit anti-p-smad2	Bioss	2% non-fat milk	1:500
Rabbit anti-Smad3	Bioss	2% non-fat milk	1:500
Rabbit anti-p-smad3	Bioss	2% non-fat milk	1:500
Rabbit anti-IgG	Abcam	2% non-fat milk	1:1000

**Table 3:** Secondary antibodies

Secondary antibody	Company
Goat anti-rabbit IgG	Zhongshan Golden Bridge Biotechnology, Beijing, China
Goat anti-mouse IgG	Zhongshan Golden Bridge Biotechnology, Beijing, China

### GFP-LC3 punctate formation assays

The adenovirus GFP-LC3 was purchased from HANBIO Biotechnology Co.Ltd. HCMECs were transfected with 20 M.O.I. of GFP-LC3 adenovirus for 12 h and then incubated under hypoxia conditions for different times. The percentage of GFP-LC3 positive cells with GFP-LC3 punctate was calculated by counting 200 cells for duplicate samples per condition. The number of GPF-LC3 punctate was calculated by counting 200 cells for duplicate samples per condition.

### Immunofluorescence staining

HCMECs were fixed in 4% paraformaldehyde (Solarbio, Beijing, China) for 15 min, permeabilized with PBS containing with 0.1% Triton-X 100 (Solarbio, Beijing, China), then blocked with 2% BSA (Solarbio, Beijing, China) at room temperature. Samples were further incubated with primary antibodies at 4°C overnight, followed by CY3–conjugated goat anti-mouse IgG (Aspen, Wuhan, China) and FITC–conjugated goat anti-rabbit IgG (Aspen, Wuhan, China) for 1 h at room temperature. Samples were counterstained with 100 μl DAPI (Solarbio, Beijing, China) for another 15 min and visualized by confocal microscopy (Nikon, Tokyo, Japan). All images were processed using Photoshop CS 8.0 software.

### Co-immunoprecipitation assay

To investigate the interaction between p62 and Snail at the endogenous level. First, HCMECs after hypoxia incubation were washed with ice-cold PBS three times before being lysed in IP lysis buffer (Solarbio, Beijing, China) containing protease inhibitor cocktail. Then, the cell lysates were centrifuged at 12000 rpm for 20 min. The supernatant was collected and incubated with anti-p62 or rabbit IgG antibody overnight at 4°C. Protein A/G-agarose beads were added for 2 h at room temperature. The beads were washed with lysis buffer for three times. Finally, the precipitated proteins were eluted and denatured in 2 ×  SDS loading buffer and analyzed by Western blot.

### Tube formation assay

The angiogenesis assays were performed as described recently [27]. Briefly, the matrigel matrix (BD, New Jersey, U.S.A.) was thawed at 4°C overnight and placed in 96-cell culture plate at 37°C for 1 h to allow the matrix solution to solidify. Then, the cells from each group were resuspended as a density of 200000 cells/ml, next were seeded in plates (50 μl cells/well), and incubated at 37°C for 6 h. Tube formation was observed under a phase-contrast microscope (Nikon, Tokyo, Japan). The tube networks were photographed from six independent fields with a microscope and were analyzed using ImageJ software.

### Statistical analysis

All data were analyzed using GraphPad Prism 7.0 software. Quantitative data were presented as mean ± S.D. of three independent experiments. The Student’s *t*test and one-way ANOVA were applied to compare the differences amongst groups. *P*<0.05 was considered statistically significant.

## Results

### EndMT was induced by hypoxia in ECs

**Figure 1:**
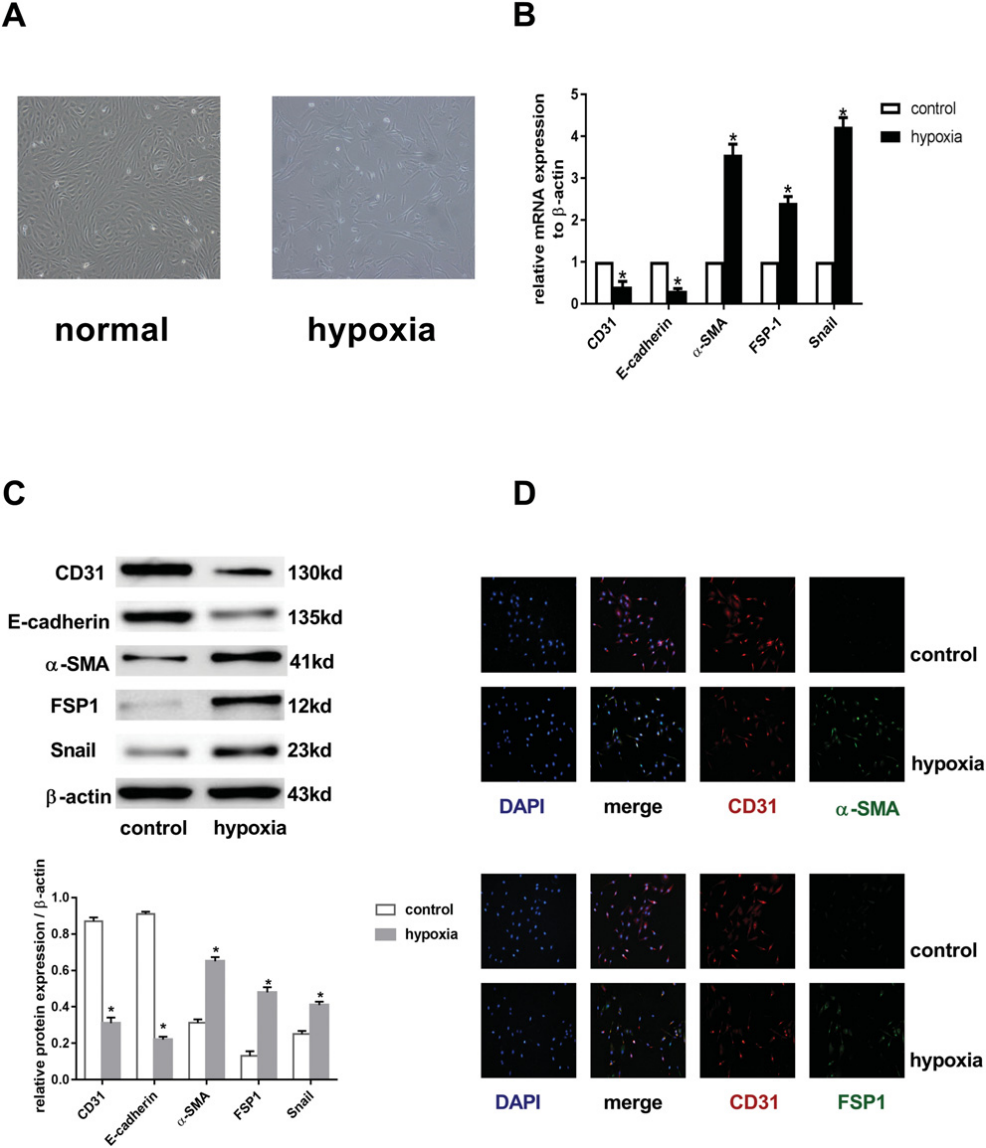
Hypoxia triggered EndMT in HCMECs (**A**) Representative phase-contrast light microscopy images showing morphological changes in HCMECs (original magnification: ×100). (**B**) RT-PCR data showing the mRNA expression levels of CD31, E-cadherin, FSP1, α-SMA, and Snail in control and hypoxia groups. Results were normalized to reference gene *β-actin* from seven independent experiments; ^*^P<0.05 compared with control group. (**C**) Western blot showing expression of CD31, E-cadherin, α-SMA, FSP1, and Snail. β-actin was used as the loading control in the control and hypoxia groups (*n*=7 independent experiments; ^*^P<0.05 compared with control group). (**D**) Representative double immunofluorescence staining images with CD31 (red) and α-SMA/FSP1 (green). Nuclei were counterstained with DAPI (blue). Acquisition of a spindle-shaped morphology upon hypoxia exposure correlated with expression of α-SMA/FSP1 and loss of CD31 (original magnification: ×200).

In order to establish the model of EndMT *in vitro*, we exposed HCMECs to hypoxia condition for 3 days. ECs changed gradually from a cobblestone phenotype to an elongated spindle-shaped morphology (Figure 1A). RT-PCR analysis (Figure 1B) showed that hypoxia decreased the expression of *CD31* and *E-cadherin* mRNAs but increased *α-SMA, FSP1*, and *Snail* mRNA levels. Compared with the control groups, cells acquired mesenchymal makers (α-SMA, FSP1, and Snail) and lost endothelial makers (CD31 and E-cadherin) (Figure 1C). Immunofluorescence results (Figure 1D) further showed that CD31^+^α-SMA/FSP1^+^ cells or CD31^−^α-SMA/FSP1^+^ cells were found in the hypoxia groups, and CD31^+^α-SMA/FSP1^−^ cells were found in the normoxia group. All these changes were time dependent (results not shown), similar as shown in our previous studies [27]. Taken together, these data demonstrated that hypoxia induced EndMT.

### Autophagic flux in ECs was increased under hypoxia

**Figure 2:**
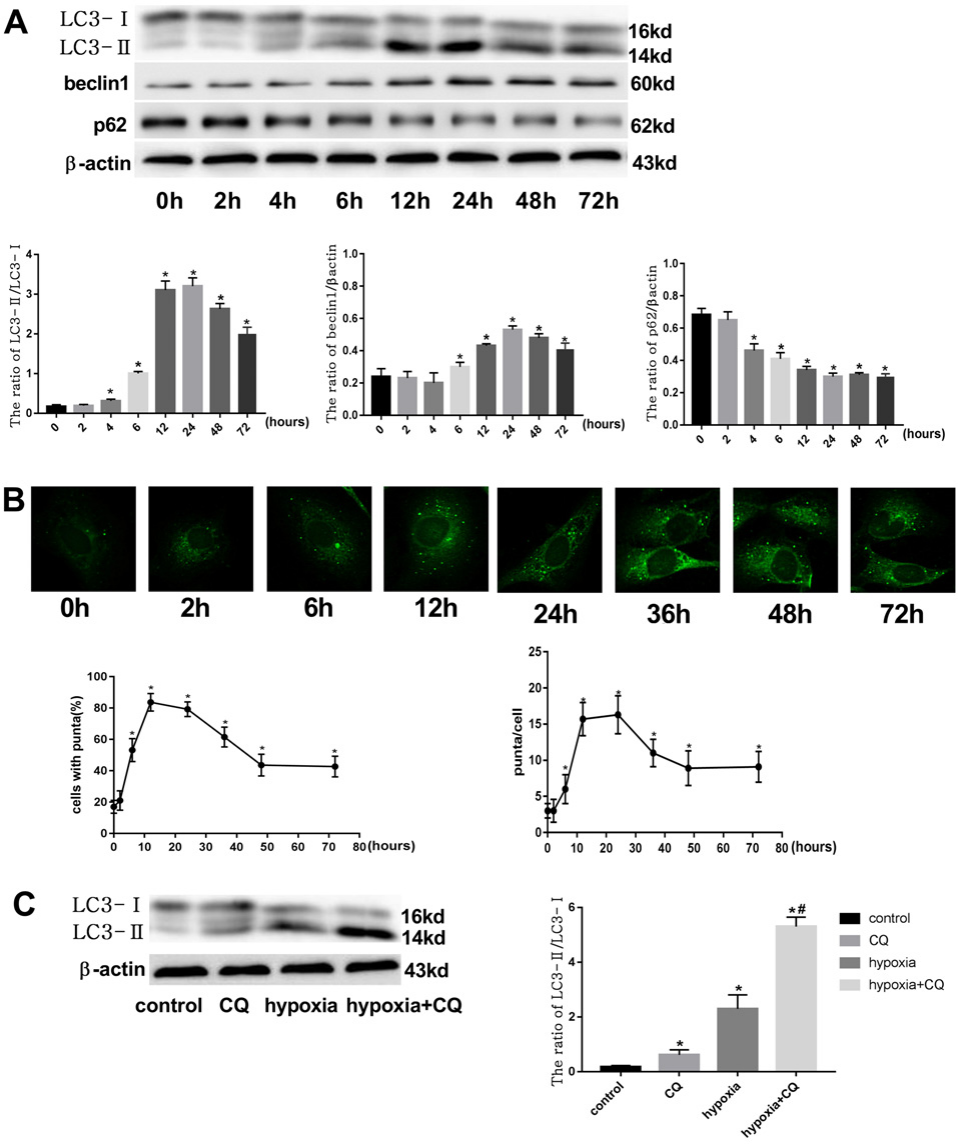
Autophagic flux in ECs increased under hypoxia (**A**) The protein expression of LC3, beclin1, and p62 was examined in HCMECs at different time points of hypoxia. β-actin was used as the loading control (*n*=7 independent experiments; ^*^P<0.05 compared with control group). (**B**) GFP-LC3 punctate formation in HCMECs at different time points of hypoxia (n=6 independent experiments; ^*^P<0.05 compared with control group). (**C**) Western blot showing the difference in LC3 expression levels between control and hypoxic conditions with or without CQ (n=7 independent experiments; ^*^P<0.05 compared with control group; ^#^*P*<0.05 compared with hypoxia group).

To detect autophagy in HCMECs under hypoxia, proteins were extracted from cells treated with 1% O_2_ for 0, 2, 4, 6, 12, 24, 48, and 72 h. As shown by Western blot, the ratio of LC3 II/I increased gradually at 4 h, reaching maximum at 12–24 h, and subsequently declined. The ratio was higher in all treatment groups other than the control group (Figure 2A). Another autophagy maker protein beclin1 expression and the number of punctate GFP-LC3 showed similar tendency with time under hypoxia conditions (Figure 2 A, 2B). In contrast, the expression of p62 protein, which is the substrate of autophagy, decreased gradually with prolongation of hypoxia time (Figure 2A). To distinguish whether the increase in LC3-II is due to autophagy induction or a block in downstream steps, LC3 turnover assays were performed. Accumulation of LC3-II increased by the treatment with CQ, an agent that impairs lysosomal acidification, even under normoxia conditions. However, the difference in LC3-II levels in the presence and absence of CQ was larger under hypoxia than normoxia conditions (Figure 2 C), indicating that autophagic flux was increased during hypoxia.

### Improving the level of autophagy partially inhibited EndMT while blocking autophagy promoted EndMT

**Figure 3:**
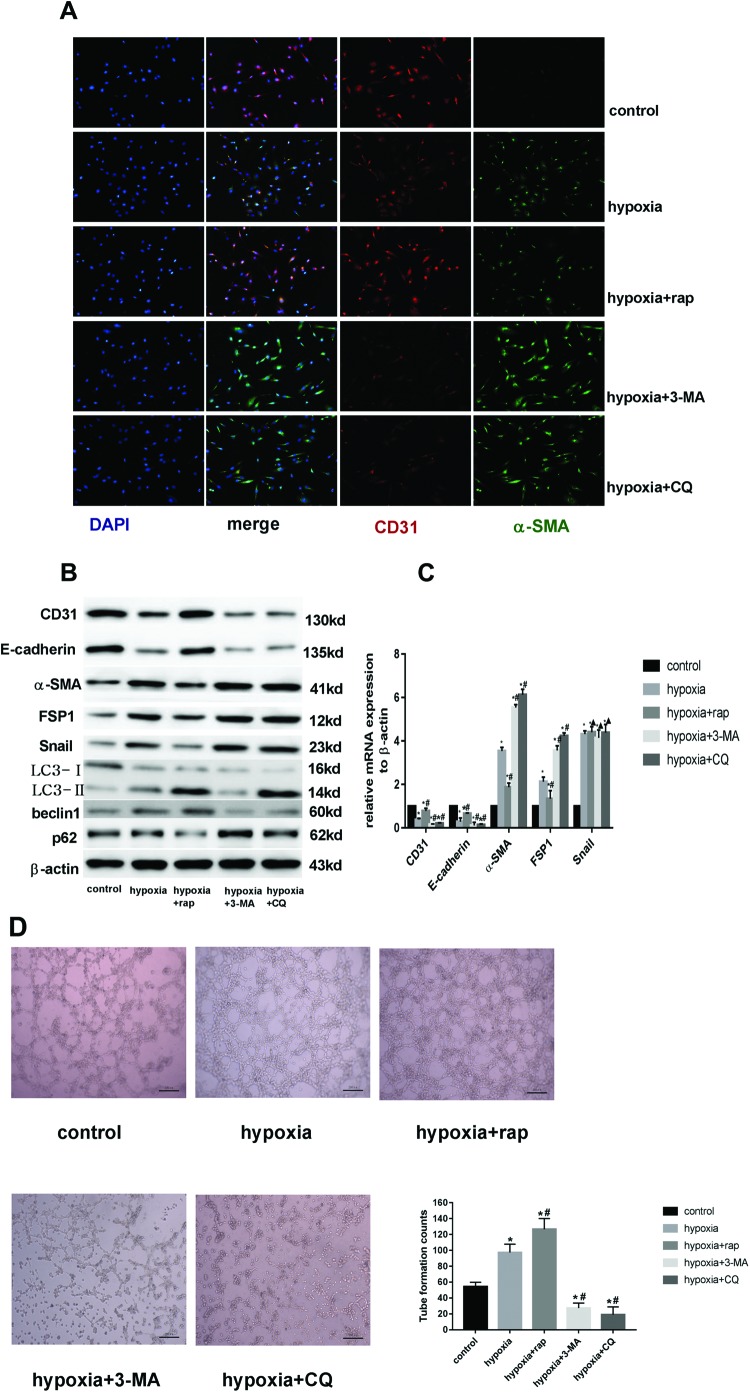
Improving the level of autophagy partially inhibited EndMT while blocking the process-promoted EndMT (**A**) Double immunofluorescence staining with antibodies to CD31 (red) and α-SMA (green). Nuclei were counterstained with DAPI (blue). Scale bars: 50 μm. Rap indicates rapamycin in all the pictures. (**B**) Western blot analysis of CD31, E-cadherin, FSP1, α-SMA, Snail, LC3, beclin1, and p62 in HCMECs from each group. β-actin was used as the loading control (*n*=7 independent experiments). (**C**) RT-PCR data showing the mRNA expression levels of CD31, E-cadherin, α-SMA, FSP1, and Snail in each group. Results were normalized to reference gene *β-actin* (*n*=7; ^*^P<0.05 compared with control group; ^#^*P*<0.05 compared with hypoxia group; ^▲^*P*>0.05 compared with hypoxia group). (**D**) Representative tube formation images observed under a phase-contrast microscope and the average of tube formation counts from six independent fields in each group. Scale bars: 200 μm (*n*=7; ^*^P<0.05 compared with control group; ^#^*P*<0.05 compared with hypoxia group)

To further investigate the effect of autophagy on the EndMT of HCMECs under hypoxia conditions, HCMECs were pretreated with rapamycin, an autophagy activator, 2 h before hypoxia exposure. Supplementation of endothelial culture medium with rapamycin decreased the number of CD31^+^α-SMA^+^ cells and CD31^−^α-SMA^+^ cells under hypoxia conditions (Figure 3A). Rapamycin partly inhibited the loss of CD31 and E-cadherin proteins and the increase in α-SMA, FSP1, and Snail proteins induced by hypoxia (Figure 3B), corresponding with the increased ratio of LC3 II/I and beclin1 expression and the decline of p62 (Figure 3B). These data indicated that further improving the level of autophagy partially inhibited EndMT in HCMECs under hypoxia conditions. In contrast, when ECs were treated with 3-MA or CQ for 2 h and incubated with hypoxia for 3 days, LC3II, beclin1, and p62 expression changes showed autophagy induced by hypoxia was blocked (Figure 3B), the expression of CD31 and E-cadherin proteins was markedly down-regulated, and the expression of mesenchymal makers and transcription factor Snail was significantly up-regulated (Figure 3B). Immunofluorescence staining showed that 3-MA and CQ markedly increased the number of CD31^−^α-SMA^+^ cells (Figure 3A). So blocking hypoxia-induced autophagy in HCMECs, EndMT was more pronounced. Similar changes in the mRNA of EndMT markers after treatment with rapamycin, 3-MA and CQ were observed, but *Snail* mRNA did not change in the presence or absence of these drugs (Figure 3C). Snail is a key transcription factor triggering EndMT. Intervening autophagy caused the corresponding changes in Snail protein, endothelial, and mesenchymal markers with no significant change in *Snail* mRNA, which suggested autophagy may not affect the synthesis of Snail protein but the degradation to influence EndMT. In addition to these, on the function study of HCMECs, we found that intervening autophagy also affected angiogenesis (Figure 3D). Compared with that in the control HCMECs, hypoxia promoted tube formation, while rapamycin further increased angiogenesis notably and 3-MA and CQ markedly suppressed the effect in HCMECs under hypoxia conditions (Figure 3D). These data indicated that autophagy may serve as a protective mechanism for promoting angiogenesis against EndMT induced by hypoxia and Snail might mediate the effect.

### Autophagy induced by hypoxia promoted Snail degradation

**Figure 4:**
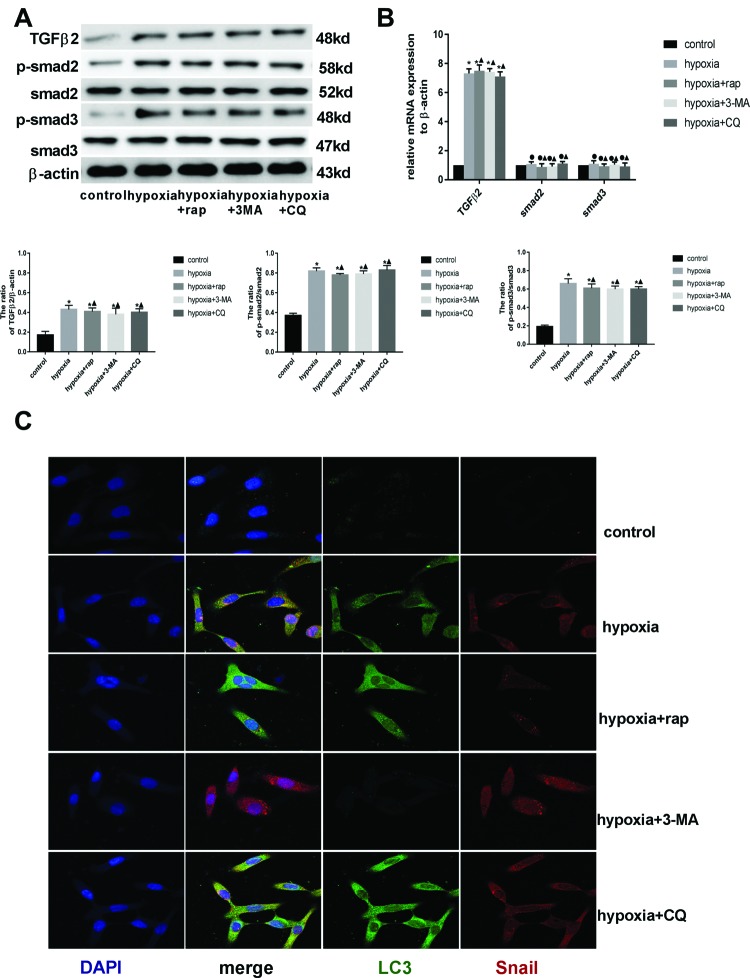
Autophagy induced by hypoxia promoted Snail degradation (**A**) Western blot analysis of TGFβ2, Smad2, p-Smad2, Smad3, and p-Smad3 in the HCMECs from each group. β-actin was used as the loading control (*n*=7; ^*^P<0.05 compared with control group; ^▲^*P*>0.05 compared with hypoxia group). (**B**) RT-PCR data showing the mRNA expression levels of TGFβ2, Smad2, and Smad3 in each group. Results were normalized to reference gene *β-actin* (*n*=7; *P<0.05 compared with control group; ^●^*P*>0.05 compared with control group; ^▲^*P*>0.05 compared with hypoxia group). (**C**) Double immunofluorescence staining with antibodies to Snail (red) and LC3 (green). Nuclei were counterstained with DAPI (blue). Scale bars: 50 μm.

**Figure 5:**
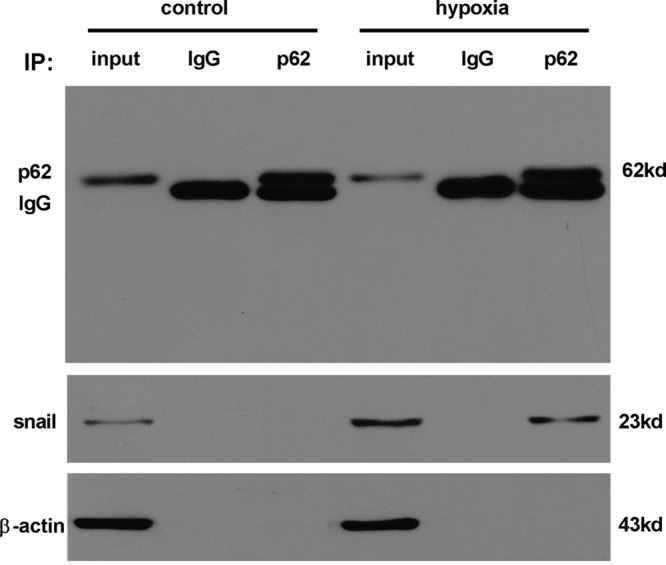
Autophagy degrades Snail in HCMECs through interaction with p62 Western blot analysis for Snail and p62 following co-immunoprecipitation of either p62 or using an isotype-matched control IgG from the same sample lysates in the control or hypoxia group. β-actin was used as the loading control (*n*=7 independent experiments).

As shown in Figure 4A,B, hypoxia up-regulated TGF-β2, p-Smad2, and p-Smad3, which suggested that hypoxia induced EndMT in HCMECs by activating TGF-β signaling pathway, consistent with recent studies [27]. Snail is the key transcription factor in the downstream of TGF-β signaling pathway. To determine whether autophagy regulates hypoxia-induced EndMT by interacting with TGF-β signaling pathway, the expression of receptors and target genes involved in TGF-β signaling pathway in each group was detected by Western blot. In the presence or absence of an autophagy activator or an autophagy inhibitor under hypoxia conditions, the expression of upstream signaling molecules in the TGF-β signaling pathway, such as TGF-β2, Smad2, p-Smad2, Smad3, and p-Smad3, did not change (Figure 4A), but the expression of downstream signaling molecules (including CD31, E-cadherin, α-SMA, FSP1, and Snail) changed (Figure 3B). In addition, rapamycin, 3-MA, or CQ did not alter the expression of *TGF-β2, Smad2, Smad3*, and *Snail* mRNA under hypoxia conditions (Figures 3C and 4B). The changes in endothelial and mesenchymal markers mRNA are described above (Figure 3C). Thus, we can see that intervening autophagy does not affect the activation of TGF-β2 signaling pathway and the expression of *Snail* mRNA under hypxia conditions, but Snail protein and EndMT markers changed. These data suggested that autophagy, a degradative pathway for intracellular proteins, modulated EndMT by interacting with Snail to promote its degradation rather than decreasing the synthesis whose mRNA change was not consistent with the protein change in the presence or absence of rapamycin, 3-MA, or CQ under hypoxia conditions (Figure 3B,C). This hypothesis was confirmed by the colocalization of LC3 and Snail in ECs of each group. Especially, a large amount of orange-colored dots were visualized in the merged images under hypoxia alone or with CQ (Figure 4C). As shown in Figure 4C, the levels of LC3 and Snail remained low in the control group, but increased significantly in response to hypoxia. Rapamycin increased LC3 and decreased Snail under hypoxia conditions; however, 3-MA decreased LC3 but increased Snail, and CQ increased both. In addition, p62 is a key regulator and substrate of autophagy and mediates the selective autophagic degradation of protein aggregates and organelles [29]. To identify the cargo protein responsible for Snail degradation, we focussed on p62 protein. We detected whether p62 interacts with Snail by performing co-immunoprecipitation experiments. As shown in Figure 5, although the very low levels of Snail protein in control cells could not verify the interaction with p62, in hypoxia-incubated cells p62 co-immunoprecipitated with Snail obviously. These data indicated that autophagy attenuated EndMT induced by hypoxia by promoting the degradation of intracellular Snail protein.

## Discussion

Although new pathophysiological mechanisms underlying myocardial fibrosis have been widely revealed, there is no effective therapy for cardiac fibrosis, probably due to its complex etiology and the limited roles of numerous intervention targets. The contribution of EndMT to myocardial fibrosis has been increasingly valued. It is estimated that 27–35% of all fibroblasts are derived from ECs undergoing EndMT in pressure-overloaded mice [5], and 26.7% in AMI rats [13]. Previous studies on cardiac EndMT mainly focussed on human coronary artery ECs (HCAECs) or human umbilical vein ECs (HUVECs) *in vivo* or *in vitro*, but less on HCMECs [5,6,17,34,38]. HCMECs dysfunction is common in ischemic cardiomyopathy, diabetic cardiomyopathy, and some patients with advanced heart failure. Microcirculation disorders reduce blood supply to the myocardium and lead to the hypoxia microenvironment in the heart, where vascular ECs may undergo EndMT and detach from the vascular bed to migrate to perivascular space or cardiac interstitium, transforming to myofibroblasts. These myofibroblasts proliferate to produce a large amount of ECM deposited in the perivascular and interstitial space, leading to functional disorder of myocardial vessels and myocardium [30], which in turn further reduces the blood supply to subsequently deteriorate the hypoxia region. Thus, EndMT prevents myocardial regeneration and suppresses angiogenesis. HCMECs and macrovascular ECs are different in the cell size and morphology, as well as the response to pathological factors [31], so in the present study we constructed a hypoxia-induced EndMT model with HCMECs *in vitro*.

Hypoxia is also a potent inducer of autophagy [32]. Inhibiting autophagy by ATG7 siRNA represses EC proliferation, migration, and angiogenesis under hypoxia conditions [33]. Several studies imply that EndMT also inhibits EC angiogenesis [27,34]. In the present study, we revealed that autophagy was involved in the regulation of EndMT and angiogenesis. *In vitro* experiments showed that hypoxia induced EndMT in HCMECs, while the accumulation of LC3 and LC3 turnover assays indicated that autophagy was activated under hypoxia conditions. Moreover, we found that rapamycin could partially reverse the occurrence of EndMT by elevating autophagy. In contrast, 3-MA or CQ could accelerate EndMT by preventing autophagy. These results indicated that loss of autophagy promoted EndMT in ECs.

Snail, a zinc finger containing transcription factor in the downstream of TGF-β signaling pathway, can be translocated into the nucleus to repress E-cadherin expression by binding E-cadherin promoter E-box and trigger EndMT or EMT [35,36]. TGFβ2 promotes EndMT through Smad-dependent and Smad-independent signaling pathways, which are necessary for promoting increased expression of the EndMT inducing transcription factor Snail. Inhibition of Snail with siRNA prevents TGFβ2-induced EndMT [37]. Hypoxia/reoxygen induces EndMT in ECs through Snail up-regulation and Snail overexpression promotes EndMT in HUVECs [38]. A recent study showed that hypoxia induced EndMT via a mechanism involving HIF-1α-induced activation of Snail. Knockdown of Snail ameliorates hypoxia-induced EndMT [17]. Our study indicated that the molecular mechanism of EndMT induced by hypoxia should be hypoxia-induced autocrine TGF-β2 elevation, which leads to Snail up-regulation triggering EndMT, which agreed with Xu et al. study [39]. Furthermore, we found that autophagy played an important role in hypoxia-induced EndMT, which has been described above; and Snail maybe a critical molecule interacting with autophagy on TGF-β signaling pathway. Under hypoxia conditions intervening autophagy obviously changed Snail protein without alterations in the expression of *Snail* mRNA, which indicated that hypoxia-induced autophagy could inhibit the occurrence of EndMT by degrading Snail not decreasing *Snail* mRNA synthesis. In the colocalization of LC3 and Snail study, a large amount of orange-colored dots were visualized in the merged images especially under hypoxia alone or with CQ. Co-immunoprecipitation experiments indicated that p62 interacted with Snail. These two results confirmed the hypothesis that autophagy inhibited EndMT by degrading Snail. Although it has been shown in breast cancer or hepatocyte EMT that autophagy inhibits EMT by promoting Snail degradation [40,41], this maybe the first study to report similar mechanism of EndMT.

Our data cannot exclude the possibility that autophagy might interact with other signaling pathways and transcriptional factors such as Notch, Twist and Slug to attenuate EndMT *in vitro*. Although specific autophagic activators and inhibitors are used in the present study, the relationship between EndMT and autophagy can be more accurately reflected by autophagy related gene knockout or overexpression in HCMECs, which is the goal of our next experiment. Since our study was performed* in vitro*, and the *in vivo* environment is more complicated, future studies are guaranteed to investigate the mechanism of EndMT in cardiac fibroblasts.

In summary, our work indicated that autophagy served as a protective mechanism against EndMT of HCMECs under hypoxia conditions by degrading the critical transcription factor Snail in the downstream of TGF-β signaling pathway *in vitro*. These findings laid the foundation for further *in vivo* experiments in future and provided a novel molecular mechanism for the research in myocardial fibrosis.

## Abbreviations

AMI: acute myocardial infarction
ATG7: autophagy related gene 7
CQ: chloroquine
EC: endothelial cell
ECM: extracellular matrix
EMT: epithelial-to-mesenchymal transition
EndMT: endothelial-to-mesenchymal transition
FSP1: fibroblast protein-1
HCMEC: human cardiac microvascular EC
HUVEC: human umbilical vein EC
LC3: microtubule-associated protein 1 light chain 3
3-MA: 3-methyladenine
M.O.I.: multiplicity of infection
PECAM-1/CD31: platelet endothelial cell adhesion molecule-1
qRT-PCR: quantitative real time polymerase chain reaction
α-SMA: α-smooth muscle actin
TGFβ: transformation growth factor beta
